# Foreign body in the tonsillary region as a complication of tonsillectomy

**DOI:** 10.1590/S1808-86942010000600022

**Published:** 2015-10-19

**Authors:** Alexandre Minoru Enoki, José Ricardo Gurgel Testa, Marcelo de Sampaio Morais, Danilo Pereira Pimentel Fernandes, Sheila Maria Cardinali Tamiso

**Affiliations:** 1 Otorhinolaryngologist, fellow in pharyngolaryngology, Medical School Clinic Hospital, São Paulo University; 2 Adjunct professor of otorhinolaryngology, Paulista School of Medicine. Head of the medical residency program in otorhinolaryngology, Paulista Hospital, São Paulo, Brazil; 3 Otorhinolaryngologist, fellow in plastic surgery of the face, Medical School Clinic Hospital, São Paulo University; 4 Physician, otorhinolaryngologist; 5 Physician, otorhinolaryngologist. Paulista Hospital of Otorhinolaryngology (Hospital Paulista de Otorrinolaringologia)

**Keywords:** needles, intraoperative complications, foreign bodies, tonsillectomy

## INTRODUCTION

Tonsillectomy is one of the most frequent surgical procedures; however, few complications of this procedure are published.^1^ The present article reports a case of a broken suturing needle in the tonsillary region during surgical hemostasis. The patient had no complaints related to this foreign body in the immediate postoperative period. Treatment consisted of a second procedure to surgically remove the needle; an image intensifier was used to locate the foreign body and facilitate its removal.

## CASE REPORT

EDD, a female patient of mixed color, aged 34 years, living in the city of São Paulo, and working as a salesperson, presented to our unit with a complaint of repeat tonsillitis (4 episodes within the past 6 months). The physical examination showed grade III tonsils, for which surgery (tonsillectomy) was recommended.

Bilateral tonsillectomy by dissection along the anatomical plane was carried out under general anesthesia and orotracheal intubation; the procedure was uneventful until the moment hemostasis was done with 2-0 catgut sutures, at which point the surgical needle broke in the lower pole of the left tonsillary region; the surgeon was unable to remove the needle during this procedure. Hemostasis of the right side was done without complications, again using 2-0 catgut sutures.

The patient presented no pain or other events related to the foreign body in the immediate postoperative period; she remained under observation and was given a prophylactic antibiotic (amoxicillin) and an analgesic (dipirone).

Antero-posterior and lateral neck radiograms, and computed tomography of the neck (Fig.) were done to locate the foreign body. It was found in the left pharyngeal palatal muscle, about 1 cm from the left carotid artery.

**Figure:**
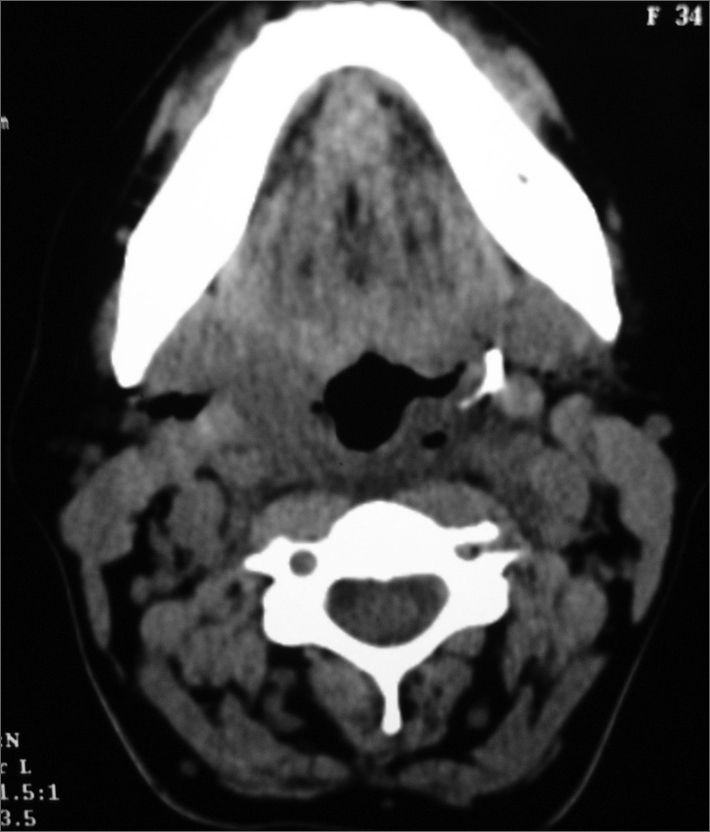
CT scan image showing the foreign body in the left tonsil lodge.

A second procedure under general anesthesia and orotracheal intubation was undertaken 48 hours later by the same team at another medical service to remove the foreign body. An image intensifier was used to help locate the foreign body, using two straight needles at an orthogonal plane (90o angle) as a guide. The left pharyngeal palatal muscle was dissected with Kelly forceps to retrieve the needle.

The patient is currently being followed-up at the outpatient unit, and after 6 months has presented no complaints or complications.

## DISCUSSION

A review of the literature until 2009 showed few reported cases of suture needles in the peritonsillar region.^2^

Foreign bodies in this region may cause several early or late postoperative complications.^3^, ^4^ Pain, edema, abscesses, and fistulae may occur in the immediate postoperative period; abscesses, chronic fistulae, bleeding, paresthesia, pseudotumors, and fibrosis may be seen in the middle or long-term.^2^

In the present case there were no complications during the 48-hour period between procedures. Authors in the literature have recommended prompt removal of such foreign bodies because of possible migration to vital anatomical structures;^4^, ^5^, ^6^, ^7^ the carotid artery is the most worrisome target.

## COMMENTS

Tonsillectomy is one of the most commonly performed procedures in the daily routine of otorhinolaryngologists. Although uncommon, intraoperative events may occur, such as broken suture needles during hemostasis of the tonsillar region; this may result in significant complication when not removed.

## References

[1] McKeown HF, Sandfer PJ (2004). A nasal foreign body detected on routine orthodontic radiographs. Br Dent J. 1998 Oct.

[2] Gündüz K, Çelenk P, Kayipmaz S (2004). Na Unusual Foreign Body (Suturing Needle) in the Tonsillar Region. J Contemp Dent Pract..

[3] al-Ruhaimi KA, Nwoku AL (1991). An unusual radiopaque image through an orthopantomogram film. Int J Oral Maxillofac Surg..

[4] Burgess JO (1988). The broken dental needle - a hazard. Spec Care Dentist..

[5] Marks RB, Carlton DM, McDonald S (1984). Management of a broken needle in the pterygomandibular space: report of case. J Am Dent Assoc..

[6] Bedrock RD, Skigen A, Dolwick MF (1999). Retrieval of a broken needle in the pterygomandibular space. J Am Dent Assoc..

[7] Zelster R, Cohen C, Casap N (2002). The implications of a broken needle in the pterygomandibular space: clinical guidelines for prevention and retrieval. Pediatr Dent..

